# A Role for Thrombospondin-1 Deficits in Astrocyte-Mediated Spine and Synaptic Pathology in Down's Syndrome

**DOI:** 10.1371/journal.pone.0014200

**Published:** 2010-12-02

**Authors:** Octavio Garcia, Maria Torres, Pablo Helguera, Pinar Coskun, Jorge Busciglio

**Affiliations:** Department of Neurobiology and Behavior, Institute for Memory Impairments and Neurological Disorders (iMIND), Center for the Neurobiology of Learning and Memory (CNLM), University of California Irvine, Irvine, California, United States of America; Brigham and Women's Hospital, Harvard Medical School, United States of America

## Abstract

**Background:**

Down's syndrome (DS) is the most common genetic cause of mental retardation. Reduced number and aberrant architecture of dendritic spines are common features of DS neuropathology. However, the mechanisms involved in DS spine alterations are not known. In addition to a relevant role in synapse formation and maintenance, astrocytes can regulate spine dynamics by releasing soluble factors or by physical contact with neurons. We have previously shown impaired mitochondrial function in DS astrocytes leading to metabolic alterations in protein processing and secretion. In this study, we investigated whether deficits in astrocyte function contribute to DS spine pathology.

**Methodology/Principal Findings:**

Using a human astrocyte/rat hippocampal neuron coculture, we found that DS astrocytes are directly involved in the development of spine malformations and reduced synaptic density. We also show that thrombospondin 1 (TSP-1), an astrocyte-secreted protein, possesses a potent modulatory effect on spine number and morphology, and that both DS brains and DS astrocytes exhibit marked deficits in TSP-1 protein expression. Depletion of TSP-1 from normal astrocytes resulted in dramatic changes in spine morphology, while restoration of TSP-1 levels prevented DS astrocyte-mediated spine and synaptic alterations. Astrocyte cultures derived from TSP-1 KO mice exhibited similar deficits to support spine formation and structure than DS astrocytes.

**Conclusions/Significance:**

These results indicate that human astrocytes promote spine and synapse formation, identify astrocyte dysfunction as a significant factor of spine and synaptic pathology in the DS brain, and provide a mechanistic rationale for the exploration of TSP-1-based therapies to treat spine and synaptic pathology in DS and other neurological conditions.

## Introduction

Down's syndrome (DS) or the triplication of chromosome 21 (trisomy 21) is the most common genetic cause of mental retardation. The cognitive deficits in patients with DS have been associated with structural changes in the architecture and alterations in the number of dendritic spines [1]. Morphological abnormalities such as unusually long spines, shorter spines, and reduced number of spines have been documented in the cortex of DS fetuses and newborns [2], [3]. Similar alterations were observed in the hippocampal formation, and additional reductions in spine number in adult DS patients have been linked to the development of Alzheimer's disease (AD) pathology [4], [5]. Spine pathology is also present in the Ts65Dn mouse model of DS, which shows decreased spine and synaptic density, and aberrant spine morphology including enlarged spines, irregular spine heads, and globular spine shapes [6]–[8]. Since dendritic spines are the primary sites of excitatory synapses, defects in spine structure and function can result in synaptic and circuit alterations leading to cognitive impairment and the progression of AD pathology in DS patients [9]. Unfortunately, there is little information available on the cellular and molecular mechanisms involved in DS spine malformation.

In recent years, a number of studies indicate that astrocytes regulate the stability, dynamics and maturation of dendritic spines [10]–[13]. In addition, astrocytes participate in the regulation of synaptic plasticity and synaptic transmission [14]–[18]. Astrocytes modulate the establishment and maintenance of synaptic contacts through the release of soluble factors such as cholesterol [19] or thrombospondins [20], or by direct physical interaction with neuronal cells [10]–[13], [21]. Our previous research indicates the presence of mitochondrial dysfunction and energy deficits in DS astrocytes leading to abnormal amyloid precursor protein (APP) processing and secretion, and to intracellular accumulation of amyloid β (Aβ) [22]. To investigate the role of astrocytes in DS spine pathology, we established a coculture system in which rat hippocampal neurons were plated on top of normal (NL) or DS astrocyte monolayers. Using this experimental paradigm, we found abnormal spine development and reduced synaptic density and activity in neurons growing on top of DS astrocytes, and identified thrombospondin 1 (TSP-1) as a critical astrocyte-secreted factor that modulates spine number and morphology. TSP-1 levels were markedly reduced in DS astrocytes and brain homogenates, and restoration of TSP-1 levels prevented spine and synaptic alterations. These results underscore the potential therapeutic use of TSP-1 to treat spine and synaptic pathology in DS and other neurodevelopmental or neurodegenerative conditions.

## Results

### Rat hippocampal neurons growing on top of human astrocytes develop extensive processes and dendritic spines

**Figure 1:**
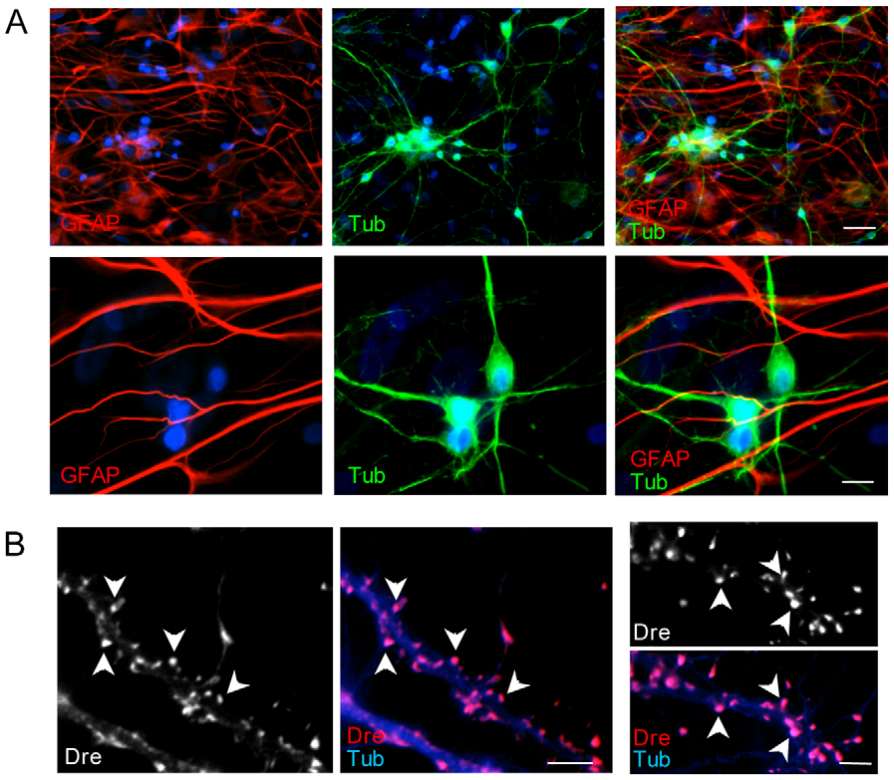
Human astrocyte/rat hippocampal neuron cocultures. Cocultures were prepared as described in the Methods section. **A**) After 21 DIV, the cells were fixed and immunostained with anti-GFAP (1∶1000, red) to visualize astrocytes, and anti-β tubulin class III (1∶1000, green) to visualize neurons. Nuclei were counterstained with Hoechst (blue). Note the significant development of neuronal processes in the cocultures. **B**) Spines (arrows) present in a typical dendritic segment visualized with anti-drebrin (1∶250, red), and anti-β tubulin class III (blue). Scale bars: A: 20 µm upper panel; 10 µm lower panel. B: 5 µm.

Previous reports have established the critical role of astrocytes in synapse formation [16], [17], [19]–[21] and regulation of dendritic spines [10]–[13]. However the role of astrocytes in DS spine pathology has not been investigated. Our previous results indicate the presence of mitochondrial dysfunction in DS astrocytes leading to alterations in protein secretion and intracellular Aβ accumulation [22]. To establish whether deficits in DS astrocyte function could be involved in spine pathology, rat hippocampal neurons were cultured on top of NL or DS astrocyte monolayers (Figure 1A). Under these conditions, rat hippocampal neurons survived well for extended periods of time and developed fast-growing axons and dendrites (Figure 1A). No differences in neuronal survival were observed between regular rat hippocampal cultures and rat hippocampal/human astrocyte cocultures (Figure S1). After 21 DIV, neurons developed numerous spines (Figure 1B) exhibiting characteristic shapes including stubby-, mushroom-, thin- and filopodium-like spines [23]. Stubby spines were especially abundant (Figure 1B). In contrast, human cortical neurons exhibited poor spine development in culture (data not shown), precluding their use for this study. Thus, to assess the capacity of DS astrocytes to sustain spine and synapse formation we utilized human cortical astrocyte/rat hippocampal neuron cocultures.

### Altered spine number and morphology in hippocampal neurons growing on top of DS astrocytes

**Figure 2:**
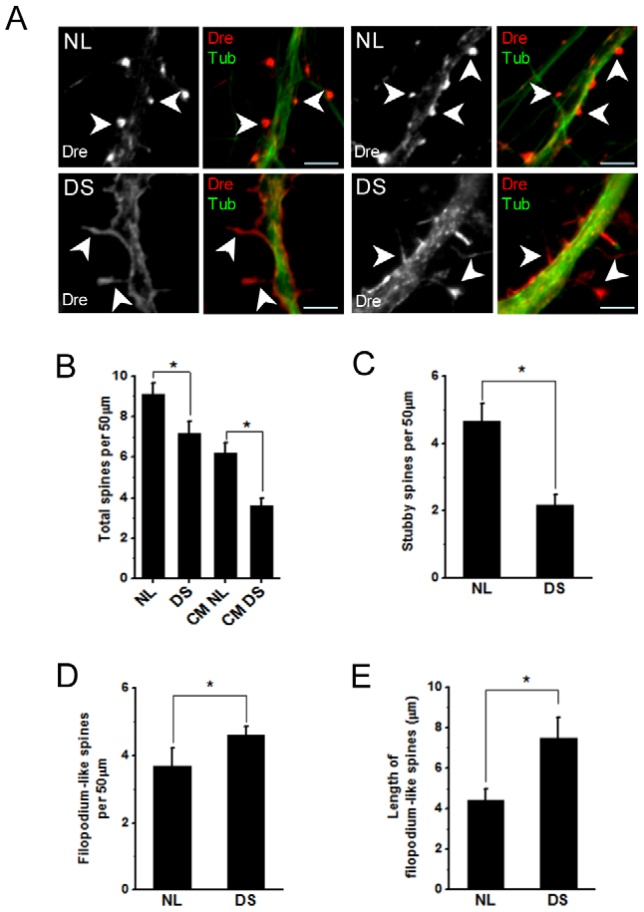
Spine number and morphology are altered in hippocampal neurons grown on top of DS astrocytes. A) Double immunofluorescence with anti-drebrin (red) and anti- β tubulin class III (green) illustrates typical spine morphologies (arrows) found in cocultures of neurons with NL and DS astrocytes respectively. The frequency of filopodium spines is significantly increased in DS cocultures. Scale bar: 5 µm. **B**) Image analysis indicates a significant reduction in the total number of spines in neurons grown on top of DS astrocytes or maintained in DS CM. In particular, this reduction affects stubby spines (**C**). Assessment of spine morphology in DS astrocyte cocultures shows a significant increase in the number (**D**) (NL 3.7±0.5; DS 4.6±0.2 SEM)**,** and length (**E**) (NL 4.4±0.5; DS 7.5±1 SEM) of filopodium spines. Spine number represents the average number of spines scored in a 50 µm dendritic segment. Data were analyzed by one-way analysis of variance (ANOVA) followed by Fisher's test. All data are expressed as mean ± SEM. *p<0.05.

We used immunofluorescence to analyze spine formation in the cocultures at 21 DIV. For simplicity, we assessed both stubby- and mushroom-like spines as “stubby”, and both thin- and filopodium-like spines as “filopodium” spines. We found a significant reduction in the total number of spines in neurons growing on top of DS astrocytes compared to neurons growing on top of NL astrocytes (NL: 9.1±0.5 per 50 µm of dendrite; DS: 7.1±0.6 per 50 µm of dendrite)(Figure 2A and B), indicating that DS astrocytes are less efficient than normal astrocytes in supporting dendritic spine formation. The reduction in spine density affected specifically stubby spines (NL: 4.6±0.5 stubby spines per 50 µm dendrite; DS: 2.1±0.3 stubby spines per 50 µm dendrite) (Figure 2C). Conversely, in DS astrocyte/hippocampal neuron cocultures we observed a significant increase in the number and length of filopodium spines (Figure 2A, D and E), which previous studies have characterized as immature spines in hippocampal neurons [24]. Similar alterations in spine number and structure are present in DS brains [2], [3], [25], suggesting that astrocyte dysfunction contributes to spine pathology in DS.

Soluble factors released by astrocytes such as cholesterol or thrombospondins have been shown to modulate spine formation and the establishment and maintenance of synaptic contacts [19], [20]. We assessed the effect of astrocyte-released factors on spine formation. Pure hippocampal cultures grown on polylysine and maintained with astrocyte conditioned medium (CM) exhibited somewhat lower spine density than their counterparts grown on top of astrocyte monolayers, indicating that physical contact with astrocytes is also relevant for spine formation (Figure 2B). Hippocampal neurons maintained in DS astrocyte CM showed a marked reduction in the number of spines compared to neurons maintained in NL astrocyte CM (NL CM: 6.2±0.5 spines per 50 µm of dendrite; DS CM: 3.6±0.3 spines per 50 µm of dendrite)(Figure 2B). Thus, soluble factors are critically involved in DS astrocyte-mediated spine alterations.

### Reduced TSP-1 levels in DS astrocytes and DS brains

**Figure 3:**
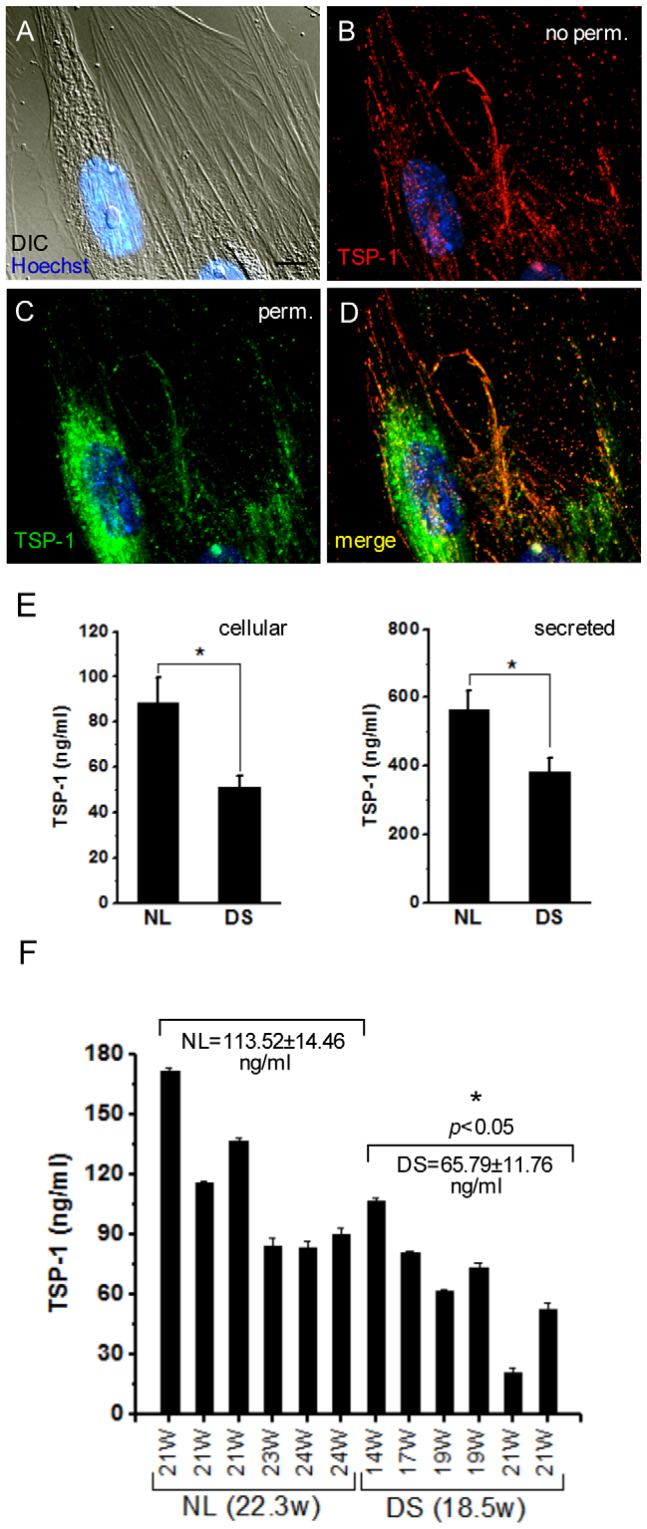
Reduced TSP-1 levels in DS astrocytes and DS fetal brain tissue. Human astocytes in culture express abundant TSP-1**. A**) Differential interference contrast microscopy (DIC) image of the field shown in B-D. **B**) Cell surface TSP-1 IF prior to permeabilization of the cells (red fluorescence). **C**) After permeabilization, the preparation was incubated again with anti-TSP-1 (green fluorescence). Note the strong perinuclear staining. **D**) Merged image of the fields shown in B and C. Nuclei were counterstained with Hoechst (blue). Scale bar: 10 µm. **E**) Measurement of TSP-1 levels in homogenates (cellular) and CM (secreted) indicates significant reductions in DS astrocytes. Homogenates: NL 88.4±11.1 ng/ml, DS 51.51±4.7 ng/ml; CM: NL 564.0±54.2 ng/ml, DS 381.9±42.0 ng/ml. **F**) TSP-1 levels were measured in NL (n = 6) and DS (n = 6) fetal cortical brain samples as described in the Methods section. Average gestational ages of NL and DS samples were 22.3 weeks and 18.5 weeks respectively. The concentration of TSP-1 was markedly reduced in DS brain samples (average values above of the bars). Data were analyzed by ANOVA followed by Fisher's test. Error bars indicate the mean ± SEM. *p<0.05.

TSP-1 is an extracellular matrix protein synthesized and released by astrocytes [26], which is highly expressed during development of the nervous system [27]. It promotes neurite outgrowth and survival [28]–[30], neuronal migration [31], [32] and synaptogenesis [20]. Recently, it has been demonstrated that TSP-1 is necessary for synaptic and motor recovery after stroke [33], suggesting that TSP-1 also participates in neuronal plasticity. Given that TSP-1 is one of the most abundant proteins secreted by human astrocytes (data not shown), and that there are marked deficits in protein secretion in DS astrocytes [22], we next analyzed TSP-1 expression and secretion in DS astrocyte cultures and brain homogenates. We found high TSP-1 expression in human astrocytes and prominent surface localization (Figure 3A-D). TSP-1 subcellular localization and staining pattern was similar in normal and DS astrocytes. Quantification of TSP-1 protein levels by ELISA demonstrated significant reductions in both DS astrocyte cell lysates and CM (Figure 3E), suggesting that reduced TSP-1 expression leads to decreased TSP-1 in DS CM. TSP-1 expression was considerably higher in fetal than in adult brain homogenates (data not shown), consistent with a significant role of TSP-1 during development [27]. A comparison of TSP-1 protein expression in fetal brain lysates showed an average reduction of 57.9% in TSP-1 levels in DS brains despite the average age of DS brain samples being almost 4 weeks younger than the average age of NL brain samples (Figure 3F). qPCR analysis of mRNA expression indicated similar mRNA levels in DS brains than in NL brains for all thrombospondin isoforms (TSP-1, -2, -3 and -4)(Figure S2), suggesting that a post transcriptional mechanism underlies the changes observed in TSP-1 in DS brains and astrocyte cultures.

### TSP-1 regulates spine morphology and prevents spine alterations in neurons grown on top of DS astrocytes

**Figure 4:**
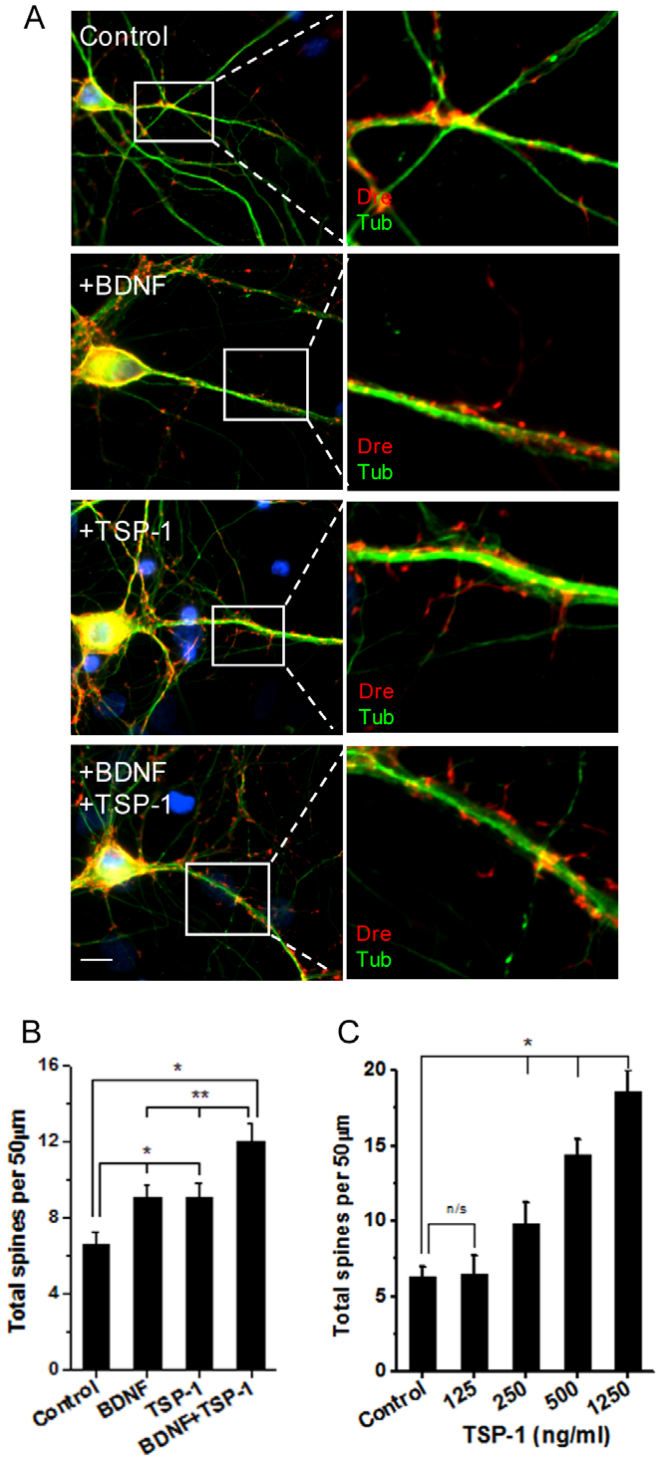
TSP-1 modulates spine development. A) Pure rat hippocampal cultures were incubated with BDNF (10 ng/ml), recombinant TSP-1 (250 ng/ml), or TSP-1+BDNF as described in the Methods section. Double immunofluorescence shows anti-Drebrin (red) and anti-β tubulin class III (green) immunostaining. Note abundant spine development with the treatments. Nuclei were counterstained with Hoechst (blue). Scale bar: 10 µm. **B**) TSP-1 and BDNF significantly enhance total spine number to similar levels. Coincubation with TSP-1+ BDNF produces a higher increase in spine number than either individual factor. Error bars indicate the mean ± SEM. *p<0.05 *vs* Control, **p<0.05 *vs* TSP-1 or BDNF. **C**) Increasing concentrations of TSP-1 induces a gradual increase in spine density in hippocampal neurons. Error bars indicate the mean ± SEM. *p<0.05.

We assessed the effect of TSP-1 on spine development in pure hippocampal cultures. Recombinant TSP-1 was added to the culture medium at day 7, and fresh TSP-1 was replenished every 3 days for 14 days. We used a TSP-1 dose of 250 ng/ml because it is in the range of the difference in the level of TSP-1 in CM between NL and DS astrocyte cultures (Figure 3E). TSP-1 increased significantly the number of spines compared to vehicle-treated cultures (control: 6.5±0.7 spines per 50 µm dendrite; TSP-1: 9.0±0.7 spines per 50 µm dendrite)(Figure 4A and B). The effect of TSP-1 on spine number was similar to the effect of BDNF, a well known inducer of spine development in hippocampal neurons [34], [35]. Combined treatment of BDNF+TSP-1 had an additive effect on spine development, suggesting that the two factors act through non-overlapping, complementary signaling pathways (Figure 4A and B). A dose-response assay indicated that concentrations of TSP-1 between 250 ng/ml to 1250 ng/ml produced a gradual and marked increase in the number of dendritic spines in pure neuronal cultures (Figure 4C). Thus, TSP-1 is a strong promoter of spine development.

**Figure 5:**
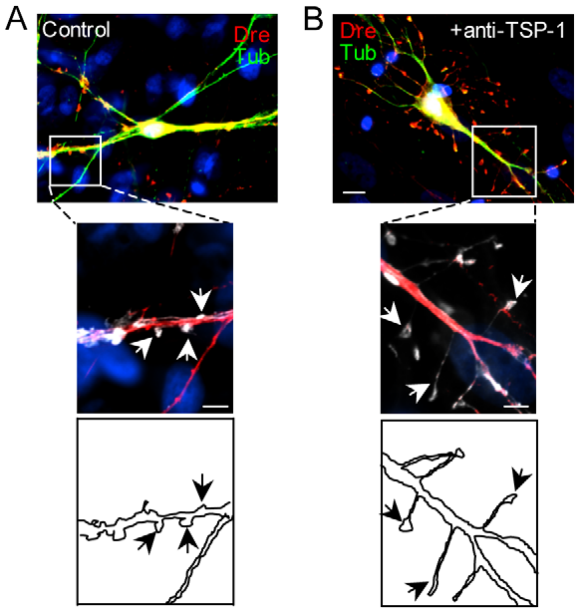
Depletion of TSP-1 markedly alters spine morphology. Cocultures of rat hippocampal neurons and NL astrocytes were incubated with a control IgG (**A**) or anti- TSP-1 (**B**). The region outlined by rectangles is shown at higher magnification in the panels below. Cocultures immunodepleted of TSP-1 exhibit dramatic changes in spine morphology including a marked increase in long filopodium-like spines (arrows) compared to control cultures, in which stubby spines predominate (arrows). Cocultures were fixed and immunofluorescence was performed with anti-drebrin (red) and anti- β tubulin class III (green) antibodies. The panels at higher magnification have been pseudocolored in red (tubulin) and white (drebrin) to facilitate the visualization of spine morphologies. Nuclei were counterstained with Hoechst (blue). Scale bars: 10 µm (upper panel), 5 µm (lower panel).

**Figure 6:**
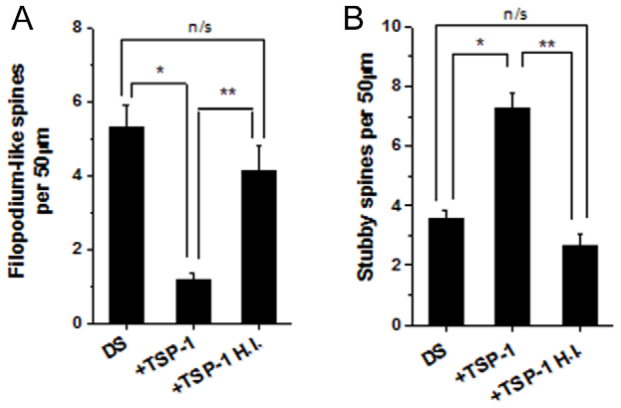
TSP-1 prevents spine alterations in neurons grown on top of DS astrocytes. Recombinant TSP-1 (250 ng/ml) was added to DS astrocyte/hippocampal neuron cocultures. TSP-1 induced a significant reduction in the number of filopodium-like spines (DS: 5.3±0.5; DS+TSP-1: 1.2±0.1)(**A**), and a marked increase in the number of stubby spines (DS: 3.5±0.3; DS+TSP-1: 7.2±0.5)(**B**). Heat-inactivated (H.I.) TSP-1 did not have any effect on spines. Data were analyzed by ANOVA followed by Fisher's test. Error bars indicate the mean ± SEM. *p<0.05, **p<0.05.

To determine whether depletion of TSP-1 is sufficient to alter spine number and morphology, NL astrocyte/hippocampal neuron cocultures were treated with anti-TSP-1 antibody to neutralize the endogenous TSP-1 secreted by NL human astrocytes. At day 7, anti-TSP-1 was added to the cultures and replenished every 3 days during 14 days. Hippocampal neurons treated with anti-TSP-1 exhibited dramatic changes in spine morphology, including a marked reduction in the density of stubby spines, and a major increase in the frequency and length of filopodium spines (Figure 5 and Figure S3). Anti-TSP-1 previously neutralized with excess TSP-1 and non-immune IgG had no effect on spine morphology (data not shown). Conversely, addition of recombinant TSP-1 to hippocampal neurons growing on top of DS astrocytes exhibited a significant reduction of filopodium spines (Figure 6A), and a marked increase in the number of stubby spines (Figure 6B). A similar effect on spines, although of slightly smaller magnitude, was observed after the addition of BDNF to the cocultures (data not shown). Heat-inactivated TSP-1 did not have any effect on spine morphology or number (Figure 6A and B). These results demonstrate that soluble TSP-1 modulates spine morphology and that reduced expression of TSP-1 in DS astrocytes leads to abnormal spine development.

### Altered spine morphology in neurons growing on top of TSP-1 KO astrocytes

**Figure 7:**
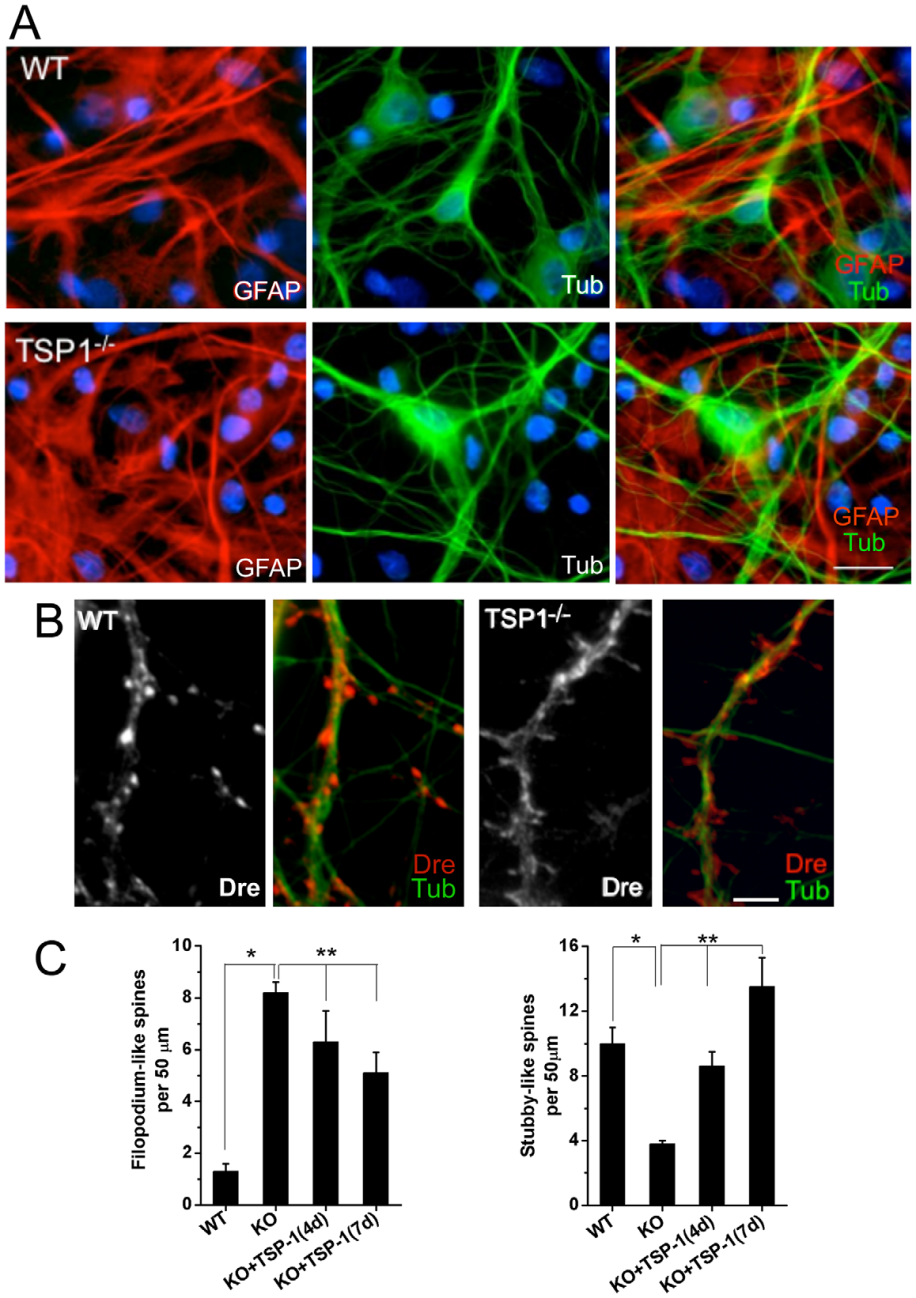
Altered spine morphology in neurons growing on top of TSP-1 KO astrocytes. Rat hippocampal neuron/mouse astrocyte cocultures were prepared as described in the Methods section. (**A**) After 21 DIV, the cultures were fixed and immunostained with anti-GFAP (1∶1000, red), an astrocytic marker, and anti-β tubulin class III (1∶1000, green), a neuronal marker. Hoechst was used for nuclear staining (blue). Similar neuronal viability and growth was observed in WT and TSP-1 KO (TSP-1^−/−^) cocultures. Scale bar: 20 µm. (**B**) Double immunofluorescence with anti-drebrin (red) and anti- β tubulin class III (green) illustrates the differences in spine morphology in neurons growing on top of WT or TSP-1 astrocytes. Note the presence of numerous filopodium spines in TSP-1 KO cocultures. Scale bar: 5 µm. (**C**) Assessment of spine morphology indicates a significant increase in filopodium spines in TSP-1 KO cocultures (KO) compared to WT cocultures. Conversely, the number of stubby spines is reduced more than 50% in TSP-1 KO cocultures. Continuous treatment with recombinant TSP-1 (250 ng/ml) during 4 days (4D) or 7 days (7D) prior to fixation at day 21 averted the spine alterations in TSP-1 KO cocultures. Spine number represents the average number of spines scored in a 50 µm dendritic segment. Data were analyzed by ANOVA followed by Fisher's test. All data are expressed as mean ± SEM. *p<0.05.

To directly address the role of TSP-1 on the regulation of spine formation, rat hippocampal neurons were grown on top of astrocyte monolayers derived from wild type (WT) or TSP-1 KO mice [36]. There were no significant differences in general morphology, viability, between both cocultures (Figure 7A). However, neurons growing on top of TSP-1 KO astrocytes exhibited a striking increase in the number of filopodium spines and a marked reduction in the number of stubby spines (Figure 7B and C). Treatment of TSP-1 KO cocultures with recombinant TSP-1 for 4 days reduced significantly the number of filopodium spines and increased the number of stubby to similar levels than WT cocultures (Figure 7C). The effect of TSP-1 was more pronounced after 7 days of treatment (Figure 7C). The spine phenotype in TSP-1 KO cocultures is reminiscent to the alterations observed in DS cocultures, providing direct evidence for a role of TSP-1 on spine formation.

### Synaptic density and the number of functional synapses are reduced in neurons grown on top of DS astrocytes

**Figure 8:**
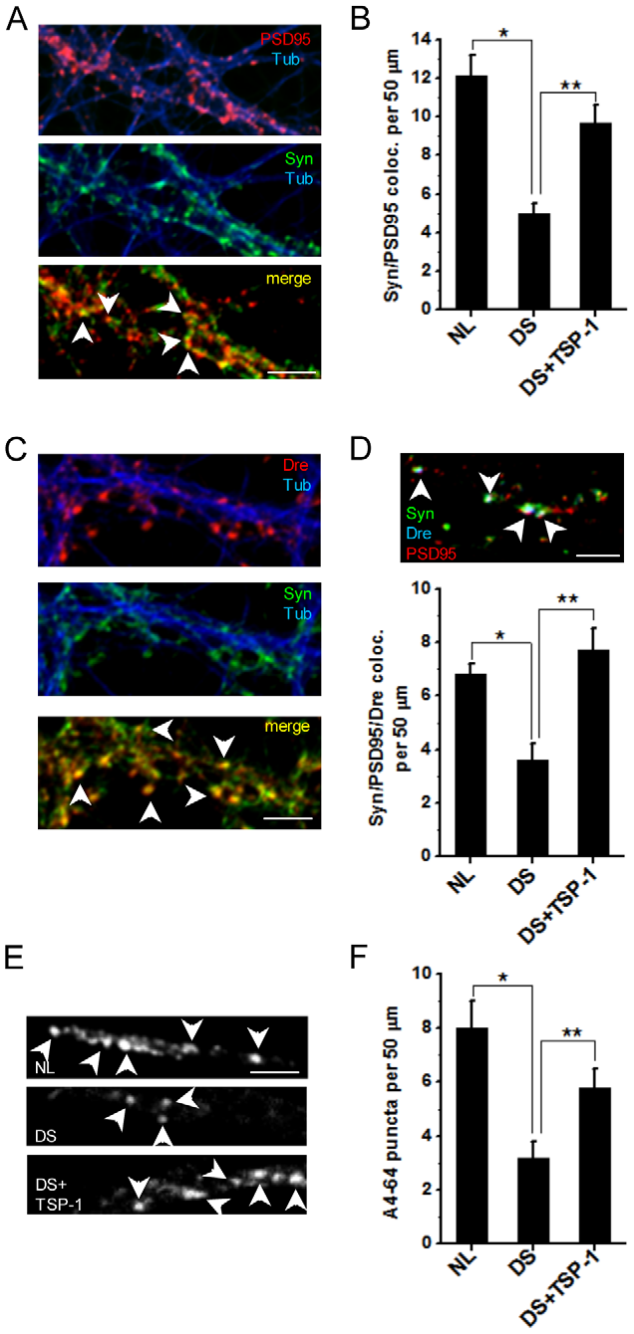
Reduced synaptic density in hippocampal neurons grown on top of DS astrocytes can be reversed by treatment with TSP-1. **A**) Colocalization of post-synaptic marker PSD-95 (1∶250, red) and pre-synaptic marker synaptophysin (1∶250, green)(arrows) was utilized to define synaptic contacts and to assess synaptic density. Neuronal processes were stained with tubulin class III (blue). **B**) Synaptic density was expressed as the number of colocalized puncta per 50 µm of dendrite in NL, DS and DS treated with 250 ng/ml TSP-1 cocultures. Error bars indicate the mean ± SEM. *p<0.05 vs cocultures of NL astrocytes, **p<0.05 vs cocultures of DS astrocytes. **C**) The colocalization of synaptophysin, drebrin and tubulin class III denotes the presence of synapses at spines (arrows). **D**) The number of synapses localized at spines was assessed by quantifying the triple colocalization of synaptophysin, PSD95 and drebrin (arrows). Error bars indicate the mean ± SEM. *p<0.05. **E**) The fluorescent cationic styryl fixable dye AM4-64, which is taken up during vesicle recycling, was used to study synaptic activity in the cocultures. AM4-64 fluorescence is shown in living NL cultures after depolarization induced by treatment with KCl (upper panel). DS cocultures present a marked decrease in AM4-64-positive vesicles after stimulation with KCl (middle panel). Treatment with TSP-1 increases synaptic activity in DS cocultures (lower panel). **F**) Quantification of AM4-64-positive puncta shows a significant reduction in DS compared to NL cocultures, which can be reverted by TSP-1 treatment. Data were analyzed by ANOVA followed by Fisher's test. All data are expressed as mean ± SEM. *p<0.05. Scale bars: 5 µm.

We investigated the effect of spine alterations and TSP-1 deficits on synapse formation and activity in DS cocultures. Synaptic density was assessed as the frequency of colocalization of the pre- and post-synaptic markers synaptophysin and PSD95 at 21 DIV [37]–[39]. Consistent with the alterations in spine morphology and density, we found a significant reduction in the number of synaptic contacts (NL: 12.1±1.1 synaptophysin/PSD95 colocalized puncta per 50 µm of dendrite; DS: 5.0±0.5% synaptophysin/PSD95 colocalized puncta per 50 µm of dendrite)(Figure 8A and B). To determine the number of synapses specifically localized in spines (Figure 8C), we measured the frequency of colocalization of synaptophysin, PSD95 and drebrin, (Figure 8D and Figure S4). Of the total number of synapses in the culture, represented by synaptophysin/PSD95 colocalization (Figure 8A and B), more than 50% were localized at dendritic spines, represented by synaptophysin/PSD95/drebrin colocalization (Figure 8C and D), indicating that spines are principal sites of synaptic formation in the cocultures. The number of synapses localized at spines was also markedly reduced in DS cocultures (Figure 7D). To study the presence of functional synapses, we assessed vesicular uptake using the fluorescent cationic styryl fixable dye AM4-64. Depolarization induced by 20 mM KCl causes rapid vesicular uptake of A4-64, allowing the visualization of recycled vesicles [37], which in this case was used as an indicator of active synapses. Depolarization induced massive vesicle recycling in both NL and DS cocultures, indicating the presence of a high number of functional synapses in the cocultures (Figure 8E). However, there was a significantly lower density of A4-64-positive vesicles in DS cocultures after stimulation with KCl (Figure 8F). These findings demonstrate that the total number of synapses, the number of synapses at spines, and the number of active synapses are all reduced in DS cocultures.

### TSP-1 increases synaptic density and activity in neurons grown on top of DS astrocytes

The ability of TSP-1 to modulate spine formation (Figure 4), to rescue spine defects associated with DS and TSP-1 KO cocultures (Figure 6 and 7), and its capacity to stimulate synaptogenesis [20], prompted us to examine the effect of TSP-1 treatment on synapse formation in neurons growing on top of DS astrocytes. Addition of 250 ng/ml TSP-1 resulted in a major increase in both total synaptic density (Figure 8B) and in the number of synapses localized at spines (Figure 8D). Restoration of TSP-1 levels also increased synaptic activity in DS cocultures as measured by A4-64 uptake (Figure 8E and F).

### Antioxidants and mitochondrial cofactors have no effect on TSP-1 expression and secretory deficits in DS astrocytes

Protein synthesis and secretory function are highly dependent on mitochondrial energy production and are particularly vulnerable to the effects of intracellular oxidative damage. For example, APP secretory deficits in DS astrocytes are directly linked to impaired mitochondrial metabolism and abnormal secretory function [22], and increased apoptosis of DS neurons can be prevented by antioxidants [40]. To establish whether reduced expression of TSP-1 in DS astrocytes was associated with oxidative stress and/or energy deficits in DS cells, we analyzed the effect of free radical scavengers, antioxidants, and energy substrates on TSP-1 expression. DS astrocytes were treated with sPBN (100 µM), trolox (100 µM), resveratrol (100 µM), nicotinamide (15 mM), nicotinamide adenine dinucleotide (β–NAD, 15 mM), creatine (5 mM) or glucose (5 mM), and TSP-1 levels were quantified 24 hr later. None of the compounds tested were able to modify TSP-1 levels inside the cells or in the CM, suggesting that neither mitochondrial dysfunction nor oxidative stress are primary factors mediating TSP-1 deficits in DS astrocytes (Figure S5).

## Discussion

Dendritic spine abnormalities have long been recognized as structural correlates of mental retardation in DS [1], [9], [41]. However, the origin and mechanisms involved in DS spine alterations are not known. Here, we show that: 1) DS astrocytes play a significant role in spine pathology and reduced synaptic density; 2) both DS brains and DS astrocytes show deficits in TSP-1 levels and secretion; 3) TSP-1 modulates dendritic spine development and morphology; and 4) TSP-1 addition reverts DS astrocyte-mediated spine and synaptic alterations.

Astrocytes are important modulators of neuronal development, metabolism, and synaptic activity [15], [18], [42], [43]. Astrocyte alterations have been linked to diverse neurological conditions such as epilepsy [44]–[46], AD [47], [48], ischemic injury [49], amyotrophic lateral sclerosis [50], [51], and neurodevelopmental disorders such as Rett's syndrome [52], [53] and fragile X syndrome [54]. However, the role of astrocytes in DS has received less attention. Astrocytes from DS fetal brain tissue and DS mouse models show increased concentration of intracellular calcium [55], [56], altered sensitivity to oxidative stress [57], deficits in mitochondrial energy metabolism [22], [58] and abnormal APP transport and secretion [22]. In addition, there is increased S100 and interleukin-1 expression in astrocytes in DS brain cortex [59], [60]. Together, these results indicate the presence of metabolic alterations in DS astrocytes that can directly affect neuronal survival and development. Supporting this notion, Nelson and colleagues demonstrated that NL neurons co-cultured with TS16 mouse astrocytes exhibit reduced cholinergic function, while TS16 neurons co-cultured with WT mouse astrocytes display normal cholinergic activity [61]. Our data indicate that neurons growing on top of DS astrocytes exhibit abnormal changes in spine morphology and density, and a marked reduction in synaptic density. Hippocampal neurons cocultured on top of DS astrocytes display a high number and longer filopodium spines, and a significant reduction in the number of stubby spines (Figure 2), reminiscent of the spine pathology described in DS infant brains [2], [3], [62]. Filopodium spines are expressed early during hippocampal [63]–[65] and cortical development [66], [67]. Although the function of filopodium spines is unclear, some studies suggest that they are associated with early stages of synaptic formation including spinogenesis, synaptogenesis and regulation of dendritic braching [24], [64], [68]. At maturity, filopodia disappear in many neurons and are replaced by stubby spines [69], [70]. Astrocytes control the maturation and dynamics of dendritic spines by physical contact with neurons [10]–[13], and can influence synapse formation through secreted factors such as cholesterol [19], thrombospondins [20] and TNF-α [71]. Thus, functional or metabolic alterations in DS astrocytes may affect normal spine development and plasticity, ultimately leading to abnormal synapse and circuit formation.

Secretory function is integral for astrocytes neuromodulatory role. Astrocytes secrete growth factors, extracellular matrix proteins, proteases, modulators of protease activity, etc [72]–[74], which regulate neuronal development and survival [75], neuritogenesis [76] and synaptogenesis [19], [20], [77]. Human astrocytes synthesize and secrete TSP-1, an extracellular matrix component involved in cell-cell and cell-matrix communication [26], [78], which is widely expressed in the developing nervous system [27]. In vitro, human astrocytes expressed high levels of TPS-1, which was detected intracellularly and at the cell surface (Figure 3A-D). TSP-1 levels were reduced in both DS astrocyte homogenates and CM, as well as in DS fetal brains, raising the possibility that in DS, TSP-1 deficits could affect critical stages of neuronal development such as neurite outgrowth [28]–[30], [79], neuronal migration [32], or synaptogenesis [20], [80]. Indeed, reduced TSP-1 activity in cocultures of NL astrocytes is sufficient to dramatically alter spine morphology (Figure 5) and synapse formation (data not shown) in hippocampal neurons. Moreover, neurons growing on top of TSP-1 KO astrocytes developed abnormal spines reminiscent to the spines in DS astrocyte cocultures. Conversely, addition of TSP-1 prevented the alterations in spine morphology and the reductions in both synaptic density and activity in neurons growing on top of DS and TSP-1 KO astrocytes. Interestingly, thrombospondin-deficient mice exhibit deficits in recovery of motor function after stroke [33], and reduced proliferation and differentiation of neuronal progenitors [81]. Thrombospondins are expressed in forebrain of human but not nonhuman primates [82] **,** further suggesting a role for thrombospondins in synaptic plasticity and human cognition. Lastly, the loss of thrombospondins also leads to craniofacial dysmorphism [83], which is a common feature of DS subjects and DS mouse models [84], [85], raising the possibility that reduced TSP-1 levels may be associated with a number of developmental anomalies present in DS.

The stimulatory effect of TSP-1 on spine formation was of similar magnitude to that of BDNF (Figure 4), pointing to a role for TSP-1 not only in synaptogenesis [20], [80], but also in spine formation. Together, TSP-1 and BDNF potentiated their effect, suggesting the presence of non-redundant, complementary pathways for TSP-1 and BDNF action on spines. In this respect, thrombospondins have been shown to act at multiple levels in different signaling pathways [86]. TSP-1 can bind and activate integrin receptors to mediate neurite outgrowth [28], and some studies suggest that TSP-1 can reorganize the actin cytoskeleton through stimulation of phosphoinositide 3-kinase (PI3-K) [87]. TSP-1 can also induce changes in cell morphology through activation of the Src family of kinases, ERK 1/2 or Rac-1 [88]. In addition, many effects of TSP-1 as inhibitor of angiogenesis and tumor growth are mediated by nitric oxide [89], p38 MAPK [90], PI3-K, FAK [91] and the CD36 receptor [92]. Recent studies show that TSP-1 can interact with the α2δ-1 gabapentin receptor [93], and with neuroligin 1 during synaptogenesis [80]. Ongoing experiments are directed to establish which signaling pathway(s) engaged by TSP-1 mediates spine formation and morphology.

Mitochondrial dysfunction and oxidative stress are major factors contributing to DS altered cellular function and survival [22], [40], [94]-[96]. In DS astrocytes, energy depletion leads to abnormal APP metabolism and altered APP secretion, both of which can be prevented by treatment with antioxidants [22]. However, antioxidants or mitochondrial cofactors did not revert TSP-1 deficits in DS astrocytes (Figure S5). One possibility is that mechanism(s) regulating TSP-1 expression such as activation of purinergic receptors [97], muscarinic receptors [74], or nitric oxide-mediated signaling [89], [98] could be altered in DS astrocytes. Alternatively, TSP-1 reduced levels in DS may be linked to the capacity of gamma interferon (IFN-γ) to inhibit both the production and secretion of thrombospondins without changing mRNA levels [99]. IFN-γ levels are elevated in both DS [100], [101] and trisomy 16 mice [102], [103]. DS individuals are hypersensitive to IFN-γ [104]–[107] and exhibit pronounced dysregulation in the levels of IFN-γ and other cytokines [108], [109]. These actions of INF-γ in DS are likely to be related to the presence of several IFN receptor genes in chromosome 21 including IFNGR2, which is one of the subunits of the IFN-γ receptor [110]. Thus, upregulated IFN-γ signaling in DS astrocytes may lead to a downregulation of TSP-1 protein levels. Furthermore, interferons also inhibit protein glycosylation [111], which is required for TSP-1 secretion. Experiments in progress are directed to understand the role of IFN-γ signaling in the post-transcriptional regulation of TSP-1 in DS.

In summary, we found metabolic alterations in DS astrocytes that cause spine pathology and reduced synaptic density in cocultured neurons. Spine and synaptic alterations were associated with reduced expression of TSP-1, which was also significantly reduced in DS brains. Finally, the identification of a novel role for TSP-1 as a strong modulator of dendritic spine development and morphology supports the exploration of TSP-1 and downstream signaling partners as therapeutic targets to treat spine pathology and cognitive impairment in DS and other neurological conditions.

## Methods

### Ethics Statement

This study is part of an ongoing research protocol approved by the Health and Hospital Corporation of the City of New York, the Albert Einstein College of Medicine Committee on Clinical Investigation and the Internal Review Board of the University of California-Irvine. The protocols for obtaining post-mortem fetal brain tissue comply with all federal and institutional guidelines with special respect for the confidentiality of the donor's identity. Written informed consent was obtained from all tissue donors.

All procedures for the generation of rat hippocampal neuronal suspensions were reviewed and approved by the Institutional Care and Use Committee (IACUC) at the University of California Irvine, protocol number 2008-2779.

### Cell Culture

Astrocyte cultures were established from postmortem NL and DS human fetal brain tissue samples as described [22]. Normal and DS fetal human brain samples are procured at the Human Fetal Tissue Repository, Albert Einstein School of Medicine (AECOM), NY, and received and processed in the Busciglio laboratory at UCI. Cortical tissue samples were dissociated into a single-cell suspension by incubation with 0.25% trypsin/40 µg/ml DNAse (Sigma, St Louis, MO) in PBS at 37°C for 30 min, and mechanically dissociated with a fire-polished glass Pasteur pipette. Cells were plated on the bottom of tissue culture dishes or on glass coverslips, and maintained in DMEM (Invitrogen, Grand Island, NY) supplemented with 10% fetal bovine serum (HyClone, Road Logan, UT). After growing to confluence, cells were subjected to two passages to generate pure astrocytic cultures. The identity and purity of the astrocyte cultures was confirmed by immunocytochemistry with anti-GFAP and anti-S100 antibodies. To generate the cocultures, hippocampi were dissected from rat newborn pups, incubated with trypsin, and triturated through a glass Pasteur pipette as described by Kaech and Banker [112]. The neurons were plated on top of NL or DS astrocyte monolayers at 100,000 cells/mm^2^. Two hr after neuronal plating, the medium was changed to Neurobasal plus N2 and B27 supplements (Invitrogen, Grand Island, NY). Partial medium changes were performed every 5 days. Cultures were maintained for 21 days to allow for the development and maturation of dendritic spines. Under these conditions, there is minimal growth of rat astrocytes in the coculture (Figure S6). Pure hippocampal cultures were maintained in NL or DS astrocyte conditioned medium (CM) obtained from astrocyte cultures. For pure hippocampal cultures, the neurons were plated on coverslips pretreated with 1 mg/ml poly-L-lysine immediately after dissociation (Sigma, St Louis, MO). Two hr after plating, the medium was switched to NL or DS CM. Partial medium changes with fresh CM were performed every 3 days. For some experiments, astrocyte monloayers were generated from the cortex of WT and TSP-1 KO [36] newborn pups following the procedures described above.

### Immunofluorescence and image analysis

Cultures were fixed with 4% paraformaldehyde/0.12 M sucrose/PBS for 15 min at 37°C, permeabilized 15 min with 0.2% Triton X-100/PBS, and blocked for 30 min in 5% bovine serum albumin/PBS. Then, the cultures were incubated with one or more of the following primary antibodies: mouse anti-drebrin, a marker of dendritic spines (1∶250, Stressegen, Ann Arbor, MI), GFAP (1∶1000, Sigma, St Louis, MO), anti-β-tubulin isotype III (1∶1000, Sigma, St Louis, MO), mouse anti-thrombospondin-1 (1∶250 Calbiochem, La Jolla, CA), anti-synaptophysin (1∶250, Calbiochem, Gibbstown, NJ), anti-PSD95 (1∶250, Abcam, Cambridge, MA), and anti S-100β (1∶500, R&D systems, Minneapolis, MN), for 1 hr at RT, followed by a 30 min incubation in fluorescent-conjugated secondary antibody (1∶500, Alexa 350, Alexa 488 and Alexa 594, Invitrogen, Eugene, OR). An Axiovert 200 inverted microscope (Zeiss, Jena, Germany) was used for examination, imaging and quantification of various parameters of spine morphology. Images were captured with a digital camera and processed using AxioVision software (Zeiss). When required, Z-stacks were captured at 500 nm intervals, processed and rendered using the Apotome imaging system (Zeiss). Spine-like protrusions were classified according to Hering and Sheng [23]. For spine assessment, dendritic spines were defined as protrusions from the surface of dendritic processes exhibiting a high concentration of drebrin [113], [114]. Morphologically, we identified 2 populations of spines: stubby-/mushroom-like spines, short, small and rounded spines with or without a short neck, and filopodium-like spines, which are typically longer, thin and lack a head. For quantification, 10 neurons per culture were selected from 3–6 separate cultures. Density and morphology of spines were scored in dendritic segments approximately 50 µm away from the cell body. Spine length was measured using the measurement tool of AxioVision. Spine length was defined as the distance from the base of the neck to the tip of the spine. To assess synaptic density, the colocalization of pre- and post-synaptic markers was performed as described [37]–[39].

### TSP-1 measurements

TSP-1 levels were determined in astrocyte cultures and fetal brain homogenates. Astrocytes were plated in 24-well plates. Cultures were washed in ice-cold PBS and collected in Eppendorf tubes in 0.1 M NaOH. The lysates were centrifuged at 14,000 rpm in an Eppendorf microcentrifuge for 30 min at 4°C, and the supernatants were used to determine the intracellular concentration of TSP-1. Aliquots of CM from the same cultures were processed to determine the level of secreted TSP-1. Samples of fetal cortex were homogenized in RIPA buffer plus protease inhibitors (Complete, Roche, Mannheim, Germany) with a Turrax homogenizer (Ika Works, Wilmington, NC), and centrifuged at 14,000 rpm for 30 min at 4°C. TSP-1 levels in the supernatant fraction were determined using a commercial ELISA kit following the vendor's instructions (R&D systems, Minneapolis, MN). For some experiments, the following compounds (purchased from Sigma, St Louis, MO) were added to pure astrocyte cultures, and TSP-1 levels were determined after 24 hr: s-PBN (100 mM), Trolox (100 µM), resveratrol (100 mM), nicotinamide (15 mM), nicotinamide adenine dinucleotide (β–NAD, 15 mM), creatine (5 mM) and glucose (5 mM).

### TSP-1 treatments

Cocultures were plated in 24-well plates and human recombinant TSP-1 (R&D system Minneapolis, MN) was added every 3 days at the indicated concentrations. Control cultures were incubated with heat-inactivated TSP-1 (100°C for 5 min). Cocultures were fixed at 21 DIV and analysis of spines was performed as described above. For some experiments, pure hippocampal cultures were treated with TSP-1 (250 ng/ml), BDNF (10 ng/ml, PeproTech Inc, Rocky Hill, NJ) or with TSP-1+BDNF.

### Depletion of TSP-1

Cocultures were plated in 24-well plates. Starting at day 7, anti-TSP-1 (1∶1000 Calbiochem, Gibbstown, NJ) was added to the cultures, and fresh anti-TSP-1 was replenished with partial medium changes every 3 days. Control cultures were treated with anti-TSP-1 previously neutralized with an excess of recombinant human TSP-1 or with non-immune rabbit serum. Cocultures were fixed at 21 DIV to assess spine density and morphology.

### Assessment of vesicle recycling

To assess activity-dependent vesicle recycling, the cultures were incubated with the fixable fluorescent probe AM4-64 (Biotium Inc, Hayward, CA) for 5 min with or without 20 mM KCl [37]. AM4-64 fluorescent density was quantified using Axiovision.

### Statistical analysis

All experiments were repeated three to six times using cultures derived from different brain specimens. Each individual experiment was performed at least in in triplicate samples. Data were analyzed by one-way analysis of variance (ANOVA) followed by Fisher's test. Results were expressed as the mean ± SEM. Significance was assessed at *p*<0.05. All results shown correspond to individual representative experiments.

## Supporting Information

Figure S1 Similar viability in neurons growing on top of normal or DS astrocytes and pure rat hippocampal cultures. Hippocampi from rat newborn pups were processed as described (Kaech and Banker, 2006). Rat hippocampal cultures were plated on coverslips precoated with poly-L-lysine. To generate cocultures, the neurons were plated on top of NL or DS astrocyte monolayers. All cultures were fixed after 21 days. Cell viability was evaluated by direct examination of neuronal morphology by a blinded operator. Neurons were visualized after immunofluorescence with anti-beta tubulin class III. Nuclei were counterstained with Hoechst. Neurons with round or oval nuclei showing light blue fluorescence and intact neuronal morphology were considered viable. Cells exhibiting condensed or fragmented nuclei and/or disrupted neuronal processes were considered dead. Fluorescent images were captured at 630X final magnification. Five fields per coverslip from 3-6 independent experiments were randomly selected for scoring of live or dead neurons. Error bars indicate the mean ± SEM. *p<0.05.
(0.68 MB TIF)

Figure S2 Similar TSP-1, -2, -3 and -4 mRNA levels in normal and DS fetal brains. TSP-1, -2, -3 and -4 mRNA levels were quantified in four 18-23 week old DS fetal brains and five age-matched controls. Quantitative real-time PCR was performed with a LightCycler 480 Real-Time PCR System utilizing LightCycler 480 SYBR Green I Master from Roche Applied Biosciences. The expression levels were normalized using 3 housekeeping genes (HKGs), Glucose 6 Phospahate Dehydrogenase (G6DH), β-Actin (Actβ), and TATA binding protein (TBP). None of the housekeeping genes were found differentially expressed between control and DS fetal brains. The graph summarizes the fold differences of each thrombospondin isoform in DS brains compared to normal brains. Each sample was run in triplicates. The primer pairs for each gene from 5- to 3-primus end are as follows: TSP1: GCTGCACTGAGTGTCACTGTC and TCAGGAACTGTGGCATTGG; TSP2: GTGCAGGAGCGTCAGATGT, and GGGTTGGATAAACAGCCATC; TSP3: AATCTCCAGTATCGATGCAATG, and GTGGCCTCC TCC TCA CAC; TSP4: CTACCGCTGTTCCTACAGC, and GAGCCTTCATAAAATCGTACCC; G6DH: GAGCCAGATGCACTTCGTG and GGGCTTCTCCAGCTCAATC; Actβ: CAACCGCGAGAAGATGAC and GTCCATCACGATGCCAGT; TBP: TGAATCTTGGTTGTAAACTTGACC and CTCATGATTACCGCAGCAAA. The thermal cycle protocol consisted of an initial heat denaturation at 95°C for 5 min, followed by 45 cycles each of denaturation at 95°C for 10 sec, annealing at 60°C for 10 sec, and an extension at 72°C for 10 sec for all primer sets. The bars represent SD.
(0.76 MB TIF)

Figure S3 Increased number of filopodium spines after TSP-1 immunodepletion in neurons grown on top of normal astrocytes. The histogram shows the number of filopodium spines per 50 µm of dendrite in control cocultures and cocultures treated with anti-TSP-1 antibody. At day 7, anti-TSP-1 was added to the culture medium and replenished every 3 days during 14 days. The cultures were fixed at day 21, and the number and type of spines was quantified as described in the Methods section. Hippocampal neurons treated with anti-TSP-1 exhibited a significant increase in the frequency and length of filopodium spines. Data were analyzed by ANOVA followed by Fisher's test. Results are expressed as the mean ± SEM. *p<0.05. The experiment was repeated using 3 different cocultures in triplicate or cuadruplicate samples. The graph corresponds to an individual representative experiment.
(0.66 MB TIF)

Figure S4 Colocalization of synaptic and spine markers. Single channel images of triple immunofluorescence showing drebrin (spine marker), PSD95 (post-synaptic marker) and synapthophysin (pre-synaptic marker). The merged image is shown in Figure 7D.
(0.64 MB TIF)

Figure S5 Antioxidants and mitochondrial cofactors have no effect on TSP-1 expression and secretion in DS astrocytes. Astrocyte cultures were treated with the designated compounds as described in the Methods section. TSP-1 levels were quantified by ELISA in soluble fractions and cellular homogenates. Sodium 4-[(tert-butylimino) methyl]benzene-3-sulfonate N-oxide (s-PBN, 100 mM); trolox (100 µM); resveratrol (Resv, 100 mM); nicotinamide (Nico, 15 mM); nicotinamide adenine dinucleotide (NAD, 15 mM); creatine (cre, 5 mM); glucose (Gluco, 5 mM). Data were analyzed by ANOVA followed by Fisher's test. The results are expressed as the mean ± SEM. Values represent the mean from 6 independent experiments. *p<0.05 vs cocultures of NL astrocytes.
(3.26 MB TIF)

Figure S6 Quantification of rat astrocytes in rat hippocampal neuron/human astrocyte cocultures. Hippocampal cell suspensions were incubated for 1 hr with fluorescent microspheres (PS-Speck, Invitrogen, Carlsbad, CA), which are rapidly taken up by viable cells. The microspheres remain in the cytoplasm and do not affect cell function or survival. Then, hippocampal suspensions were plated on top of human astrocyte monolayers, cultured for 21 days, fixed, counterstained with Hoechst, and processed for image analysis. A) DIC and fluorescence image of a microscopic field in which fluorescent microspheres are apparent in one cell (arrow, putative rat astrocyte) and absent in the other three cells in the field (putative human astrocytes). Nuclei were stained with Hoechst. Scale bar: 10 µm. B) Total number of astrocytes in the culture was assessed by scoring astrocyte nuclei, which are easily distinguishable because of their size (3 to 5 times larger than neuronal nuclei). The number of rat astrocytes was assessed by counting astrocyte cells containing fluorescent microspheres in the cytoplasm. Rat astrocytes represented approximately 18% of the total number of astrocytes in the coculture. Data were analyzed by ANOVA followed by Fisher's test. *p<0.05.
(2.48 MB TIF)
