# Supplementary figures and images for: A Role for Thrombospondin-1 Deficits in Astrocyte-Mediated Spine and Synaptic Pathology in Down's Syndrome
Source: PLoS One. 2010 Dec 2;5(12):e14200. doi: 10.1371/journal.pone.0014200 (PMC2996288; doi:10.1371/journal.pone.0014200)

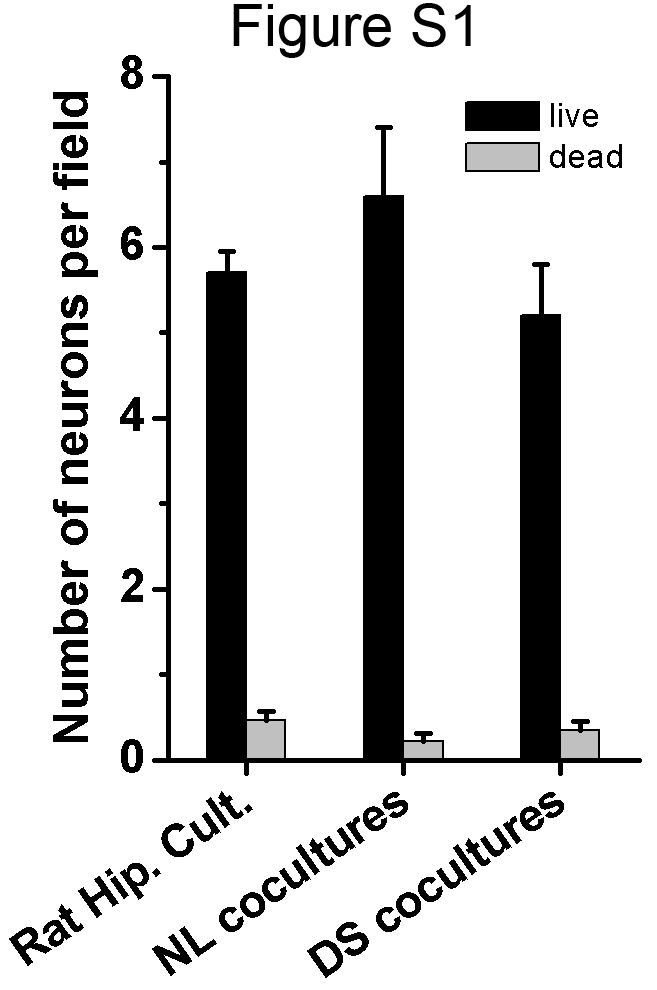

Supplement: Figure S1 — Similar viability in neurons growing on top of normal or DS astrocytes and pure rat hippocampal cultures. Hippocampi from rat newborn pups were processed as described (Kaech and Banker, 2006). Rat hippocampal cultures were plated on coverslips precoated with poly-L-lysine. To generate cocultures, the neurons were plated on top of NL or DS astrocyte monolayers. All cultures were fixed after 21 days. Cell viability was evaluated by direct examination of neuronal morphology by a blinded operator. Neurons were visualized after immunofluorescence with anti-beta tubulin class III. Nuclei were counterstained with Hoechst. Neurons with round or oval nuclei showing light blue fluorescence and intact neuronal morphology were considered viable. Cells exhibiting condensed or fragmented nuclei and/or disrupted neuronal processes were considered dead. Fluorescent images were captured at 630X final magnification. Five fields per coverslip from 3-6 independent experiments were randomly selected for scoring of live or dead neurons. Error bars indicate the mean ± SEM. *p<0.05. (0.68 MB TIF) [file pone.0014200.s001.tif]

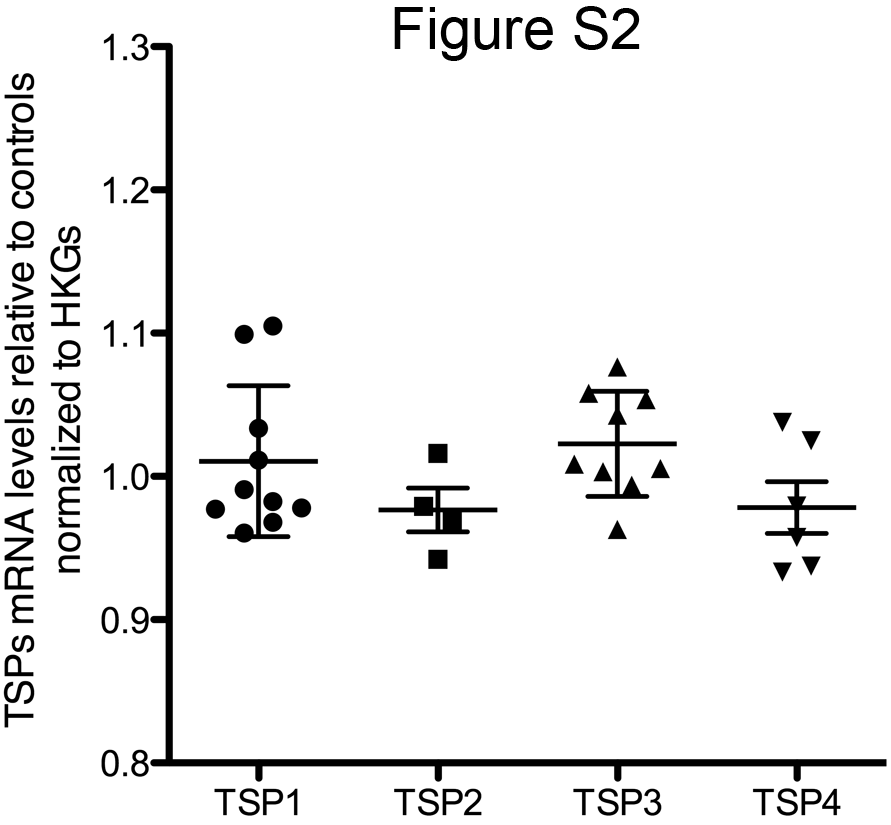

Supplement: Figure S2 — Similar TSP-1, -2, -3 and -4 mRNA levels in normal and DS fetal brains. TSP-1, -2, -3 and -4 mRNA levels were quantified in four 18-23 week old DS fetal brains and five age-matched controls. Quantitative real-time PCR was performed with a LightCycler 480 Real-Time PCR System utilizing LightCycler 480 SYBR Green I Master from Roche Applied Biosciences. The expression levels were normalized using 3 housekeeping genes (HKGs), Glucose 6 Phospahate Dehydrogenase (G6DH), β-Actin (Actβ), and TATA binding protein (TBP). None of the housekeeping genes were found differentially expressed between control and DS fetal brains. The graph summarizes the fold differences of each thrombospondin isoform in DS brains compared to normal brains. Each sample was run in triplicates. The primer pairs for each gene from 5- to 3-primus end are as follows: TSP1: GCTGCACTGAGTGTCACTGTC and TCAGGAACTGTGGCATTGG; TSP2: GTGCAGGAGCGTCAGATGT, and GGGTTGGATAAACAGCCATC; TSP3: AATCTCCAGTATCGATGCAATG, and GTGGCCTCC TCC TCA CAC; TSP4: CTACCGCTGTTCCTACAGC, and GAGCCTTCATAAAATCGTACCC; G6DH: GAGCCAGATGCACTTCGTG and GGGCTTCTCCAGCTCAATC; Actβ: CAACCGCGAGAAGATGAC and GTCCATCACGATGCCAGT; TBP: TGAATCTTGGTTGTAAACTTGACC and CTCATGATTACCGCAGCAAA. The thermal cycle protocol consisted of an initial heat denaturation at 95°C for 5 min, followed by 45 cycles each of denaturation at 95°C for 10 sec, annealing at 60°C for 10 sec, and an extension at 72°C for 10 sec for all primer sets. The bars represent SD. (0.76 MB TIF) [file pone.0014200.s002.tif]

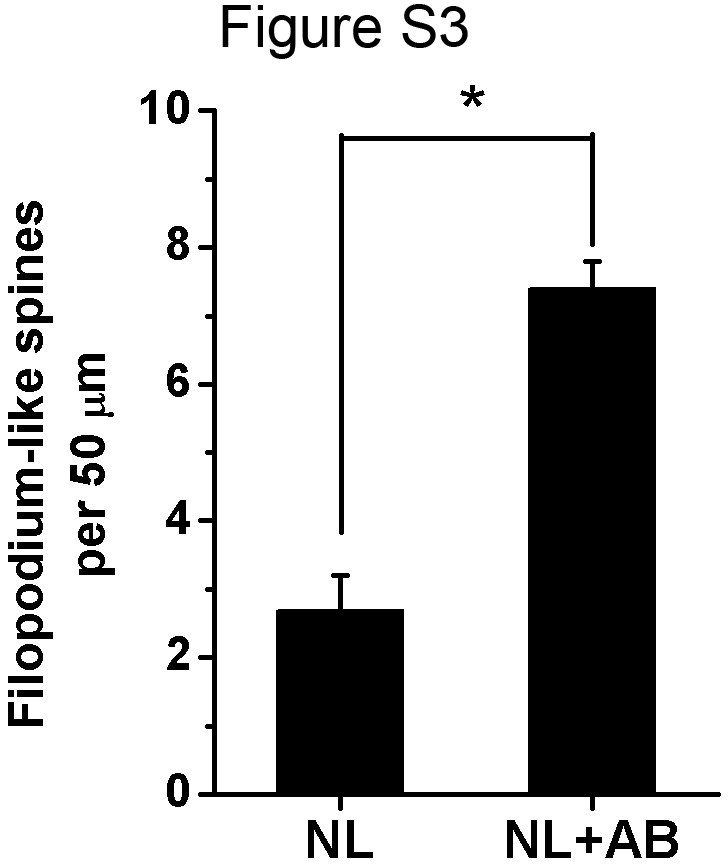

Supplement: Figure S3 — Increased number of filopodium spines after TSP-1 immunodepletion in neurons grown on top of normal astrocytes. The histogram shows the number of filopodium spines per 50 µm of dendrite in control cocultures and cocultures treated with anti-TSP-1 antibody. At day 7, anti-TSP-1 was added to the culture medium and replenished every 3 days during 14 days. The cultures were fixed at day 21, and the number and type of spines was quantified as described in the Methods section. Hippocampal neurons treated with anti-TSP-1 exhibited a significant increase in the frequency and length of filopodium spines. Data were analyzed by ANOVA followed by Fisher's test. Results are expressed as the mean ± SEM. *p<0.05. The experiment was repeated using 3 different cocultures in triplicate or cuadruplicate samples. The graph corresponds to an individual representative experiment. (0.66 MB TIF) [file pone.0014200.s003.tif]

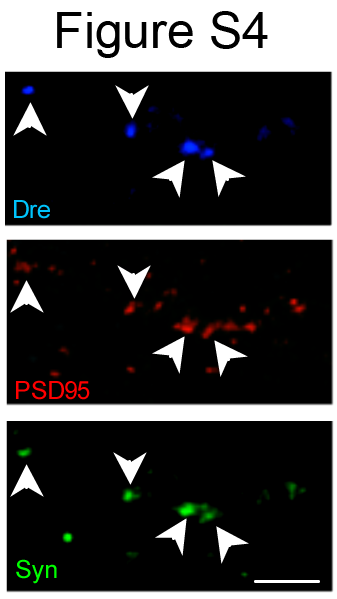

Supplement: Figure S4 — Colocalization of synaptic and spine markers. Single channel images of triple immunofluorescence showing drebrin (spine marker), PSD95 (post-synaptic marker) and synapthophysin (pre-synaptic marker). The merged image is shown in Figure 7D. (0.64 MB TIF) [file pone.0014200.s004.tif]

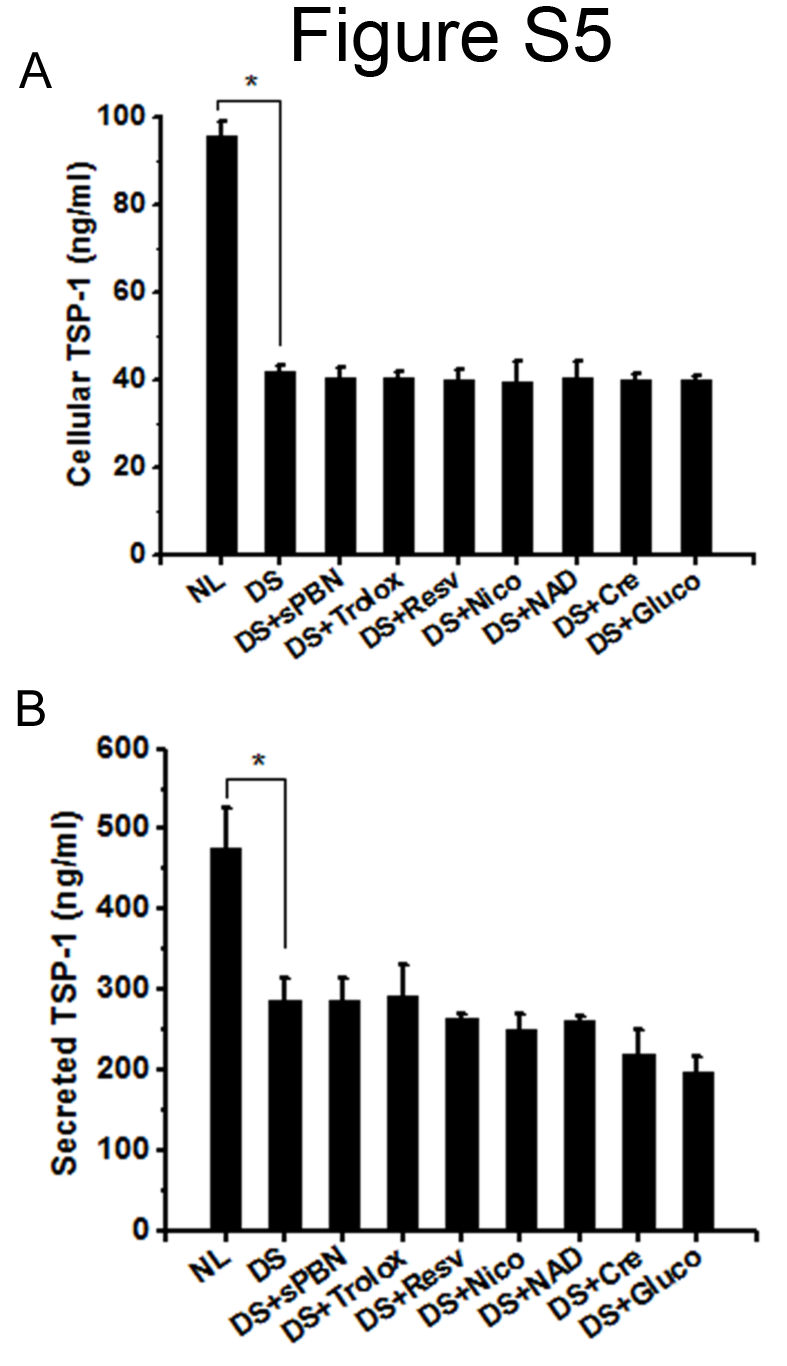

Supplement: Figure S5 — Antioxidants and mitochondrial cofactors have no effect on TSP-1 expression and secretion in DS astrocytes. Astrocyte cultures were treated with the designated compounds as described in the Methods section. TSP-1 levels were quantified by ELISA in soluble fractions and cellular homogenates. Sodium 4-[(tert-butylimino) methyl]benzene-3-sulfonate N-oxide (s-PBN, 100 mM); trolox (100 µM); resveratrol (Resv, 100 mM); nicotinamide (Nico, 15 mM); nicotinamide adenine dinucleotide (NAD, 15 mM); creatine (cre, 5 mM); glucose (Gluco, 5 mM). Data were analyzed by ANOVA followed by Fisher's test. The results are expressed as the mean ± SEM. Values represent the mean from 6 independent experiments. *p<0.05 vs cocultures of NL astrocytes. (3.26 MB TIF) [file pone.0014200.s005.tif]

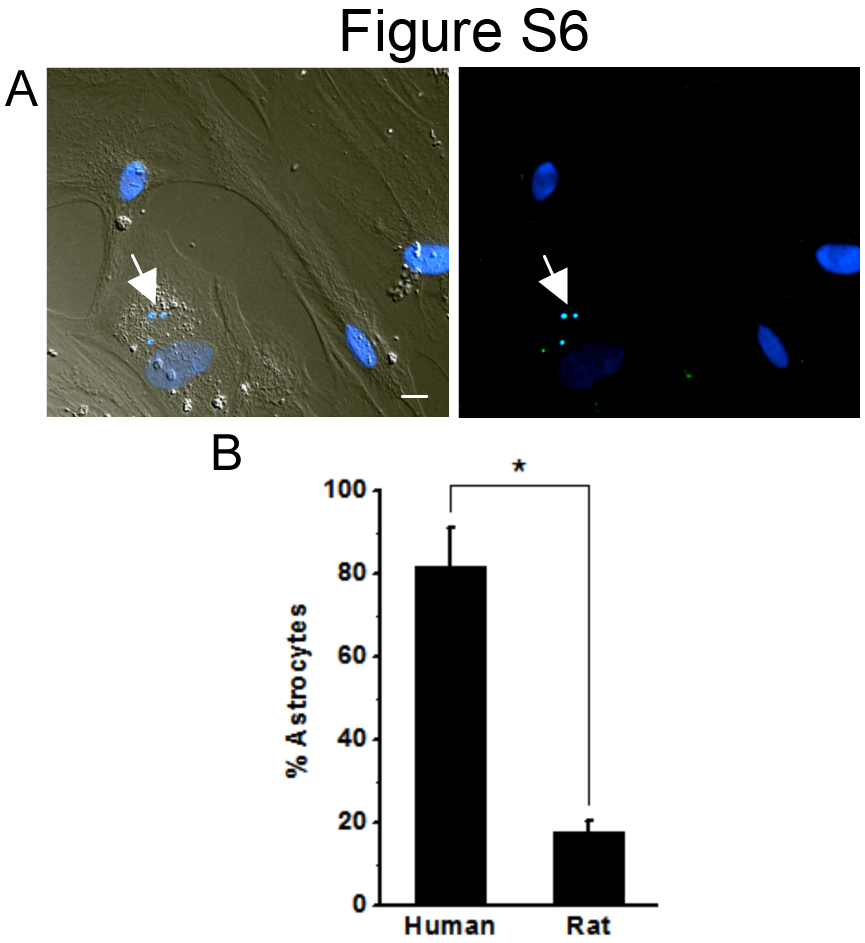

Supplement: Figure S6 — Quantification of rat astrocytes in rat hippocampal neuron/human astrocyte cocultures. Hippocampal cell suspensions were incubated for 1 hr with fluorescent microspheres (PS-Speck, Invitrogen, Carlsbad, CA), which are rapidly taken up by viable cells. The microspheres remain in the cytoplasm and do not affect cell function or survival. Then, hippocampal suspensions were plated on top of human astrocyte monolayers, cultured for 21 days, fixed, counterstained with Hoechst, and processed for image analysis. A) DIC and fluorescence image of a microscopic field in which fluorescent microspheres are apparent in one cell (arrow, putative rat astrocyte) and absent in the other three cells in the field (putative human astrocytes). Nuclei were stained with Hoechst. Scale bar: 10 µm. B) Total number of astrocytes in the culture was assessed by scoring astrocyte nuclei, which are easily distinguishable because of their size (3 to 5 times larger than neuronal nuclei). The number of rat astrocytes was assessed by counting astrocyte cells containing fluorescent microspheres in the cytoplasm. Rat astrocytes represented approximately 18% of the total number of astrocytes in the coculture. Data were analyzed by ANOVA followed by Fisher's test. *p<0.05. (2.48 MB TIF) [file pone.0014200.s006.tif]
